# Alkyl Phenols and Diethylhexyl Phthalate in Tissues of Sheep Grazing Pastures Fertilized with Sewage Sludge or Inorganic Fertilizer

**DOI:** 10.1289/ehp.7469

**Published:** 2005-01-20

**Authors:** Stewart M. Rhind, Carol E. Kyle, Gillian Telfer, Elizabeth I. Duff, Alistair Smith

**Affiliations:** ^1^Macaulay Institute, Craigiebuckler, Aberdeen, United Kingdom; ^2^Biomathematics and Statistics Scotland, Macaulay Institute, Craigiebuckler, Aberdeen, United Kingdom

**Keywords:** alkylphenol, bioaccumulation, diethylhexyl phthalate, pasture, sewage sludge, sheep, tissue

## Abstract

We studied selected tissues from ewes and their lambs that were grazing pastures fertilized with either sewage sludge (treated) or inorganic fertilizer (control) and determined concentrations of alkylphenols and phthalates in these tissues. Mean tissue concentrations of alkylphenols were relatively low (< 10–400 μg/kg) in all animals and tissues. Phthalates were detected in tissues of both control and treated animals at relatively high concentrations (> 20,000 μg/kg in many tissue samples). The use of sludge as a fertilizer was not associated with consistently increased concentrations of either alkylphenols or phthalates in the tissues of animals grazing treated pastures relative to levels in control animal tissues. Concentrations of the two classes of chemicals differed but were of a similar order of magnitude in liver and muscle as well as in fat. Concentrations of each class of compound were broadly similar in tissues derived from ewes and lambs. Although there were significant differences (*p* < 0.01 or *p* < 0.001) between years (cohorts) in mean tissue concentrations of both nonylphenol (NP) and phthalate in each of the tissues from both ewes and lambs, the differences were not attributable to either the age (6 months or 5 years) of the animal or the duration of exposure to treatments. Octylphenol concentrations were generally undetectable. There was no consistent cumulative outcome of prolonged exposure on the tissue concentrations of either class of pollutant in any ewe tissue. Mean tissue concentrations of phthalate were higher (*p* < 0.001) in the liver and kidney fat of male compared with female lambs. We suggest that the addition of sewage sludge to pasture is unlikely to cause large increases in tissue concentrations of NP and phthalates in sheep and other animals with broadly similar diets and digestive systems (i.e., domestic ruminants) grazing such pasture.

Since the ban in both the United States and Europe on the disposal of sewage sludge at sea, increasing amounts of sewage sludge are likely to be applied to farm land, including pasture grazed by ruminants (Commission of the European Communities 1994; Swanson et al. 2004). Compared to normal environmental levels in soil, water, and air, sludge contains relatively large amounts of both inorganic pollutants, such as heavy metals, and organic pollutants such as alkylphenols, phthalates, polychlorinated biphenyls (PCBs), and organochlorine pesticides (Brunner et al. 1988; Webber and Lesage 1989). Some of these compounds have endocrine-disrupting properties (Crisp et al. 1998; Toppari et al. 1996) and therefore are potentially hazardous to the health of farm animals grazing the treated pasture; the performance of grazing ruminants can be compromised by excessive exposure to environmental endocrine-disrupting compounds (EDCs) (Meijer et al. 1999). Humans may also be affected through the consumption of products derived from animals grazing contaminated vegetation. Food, particularly animal-derived products (e.g., meat, milk, cheese), is thought to represent the most important source of human exposure to many organic pollutants [Hallikainen and Vartiainen 1997; Ministry of Agriculture, Fisheries and Food (MAFF) 1997]. Knowledge of animal tissue concentrations is important for understanding the potential risk to animal health and performance as well as the risk to human health.

The formulation of sustainable practices involving recycling sludges to pasture requires a knowledge of the patterns of accumulation of EDCs in the tissues of animals grazing the pasture and of the factors that may determine the rate of accumulation, such as the class of pollutant, duration of exposure, type (e.g., grass or milk) and amount of food consumed by the animals reared on the pasture, and the age of the animal. EDCs are not generally assimilated systemically by plants (Petersen et al. 2003), but exposure of animals through the ingestion of soil or surface-contaminated herbage is possible after sludge is applied. Tissue concentrations generally represent an index of total exposure (dose × time) of the animal to these substances, but rates of accumulation are also modified by rates of uptake from the digestive tract or bloodstream and excretion or degradation.

Heavy metals (Hill et al. 1998a, 1998b) and organic pollutants such as PCBs (Fries 1996) can accumulate in the tissues of many types of animal, including domestic ruminants, after exposure to sewage sludge. Compounds such as alkylphenols and phthalates are sometimes disregarded in considerations of risk because, on the basis of their chemical structure, it is considered that these compounds are readily degraded in the environment (Jianlong et al. 2004; Smith 1995). Alternatively, it has been suggested that such compounds are degraded in the digestive system of animals ingesting them or that they are not absorbed from the gut and therefore unlikely to accumulate in tissues (Fries 1996). Consequently, alkylphenols and phthalates have been little studied, and most assessments of risk are based on simple empirical models or mathematical models, particularly with respect to terrestrial species (Scott-Fordsmand and Krogh 2004; Brooke et al. 1991).

Studies involving rats have shown that most orally administered diethylhexyl phthalate (DEHP) is degraded in the intestine to monoethylhexyl phthalate (MEHP) (Heindel et al. 1989; Koch et al. 2003; White et al. 1980); secondary oxidative metabolites derived from MEHP are biologically active (Grasso et al. 1993). However, the patterns of metabolism of EDCs differ between species (Albro and Lavenhar 1989; Watkins and Klaassen 1986). Whereas almost all DEHP may be degraded in the rat gut, significant quantities accumulate in the tissues of sheep (Boerjan et al. 2002). This suggests that in sheep ingested DEHP is not completely degraded in the gastrointestinal tract before reaching the bloodstream or that significant absorption occurs by other routes such as through the lungs (Albro and Lavenhar 1989).

In this study we investigated representatives of two groups of known EDCs, alkylphenols and phthalates (Harris et al. 1997; Lee et al. 1999; White et al. 1994). We used the practice of recycling sludge, in which large amounts of the selected EDCs are known to be present (approximately 100 mg/kg dry matter; Brunner et al. 1988; Webber and Lesage 1989) to investigate factors affecting tissue accumulation of these two groups of chemically different compounds. The primary objective was to determine the effect of enhanced exposure to EDCs (via ingested soil, contaminated vegetation, or inhalation of volatile components) on concentrations of these pollutants in the tissues of sheep. Secondary objectives were to assess the patterns of accumulation in tissues with contrasting functions and properties (liver, fat, muscle) and to determine the effects of year, duration of exposure, animal age, diet (milk or herbage), and sex of the individual on tissue concentrations.

## Materials and Methods

### Experimental design and application of sludge.

We conducted the experiment at the Macaulay Institute’s research station at Hartwood, Scotland (56° N), approximately 20 miles east of the industrialized city of Glasgow. All animals used in the study were managed according to conventional practice and treated humanely at all times.

In each year of the 3-year study, we used approximately 30 breeding ewes, 2–6 years of age, and 36 of their offspring of that year (18 male; 18 female). They were allocated randomly to one of two similar groups and maintained on the same pastures (three replicate plots per treatment) throughout the study, at normal stocking rates (3.5 ewes/ha), and we added animals to the group during periods of rapid pasture growth to ensure that the sward height was maintained at an appropriate level. Commencing in mid-summer of year 1, we applied liquid, digested sewage sludge twice annually (early spring and mid-summer) to one part of the pasture until five separate applications had been made (summer of year 3). For each application, sludge was applied at a rate of 2.25 tons of dry matter/ha to the treated pasture, using a pivot irrigation system, so that > 95% of the surface area was covered by the liquid sludge in a series of adjacent, sometimes overlapping, circles. We maintained one group of study animals (treated) on these pastures. The rate of sludge application used resulted in the application of approximately 225 kg nitrogen/ha/year, which was consistent with normal rates of nitrogen application. Patterns of sludge application to land differ with time and place, but at the time the study was conducted, the patterns of application of sludge were consistent with UK regulations and normal farming practice. However, they were also designed to result in the maximum rate of contamination of the topsoil and the maximum likely risk of exposure of grazing animals to EDCs through their food. A second, similar area of pasture was treated with an equivalent annual amount of nitrogen in the form of an inorganic fertilizer, and the second group of study animals (control) was maintained on this area throughout the study.

Throughout the study, the only material ingested by the animals was forage growing on the experimental plots and water. The animals were not fed supplements or food additives of any sort. The only drugs applied were those used in conventional animal husbandry and routine veterinary care.

Treated animals were not allowed to graze the pasture until a minimum of 3 weeks after the sludge application, as prescribed by legislation (UK Parliament 1989). This was achieved by dividing the sludge-treated pasture into two similar parts. While one part was treated, the animals grazed the other half. After the 3-week period had elapsed, the process was repeated, with the animals grazing the previously treated area. After a further 3 weeks, the animals were given access to the whole area. Treatment commenced in July of the first year when lambs were 2 months of age. Thereafter, the ewes and their offspring for each of the years of the study were maintained at all times on the treatments to which they had been allocated.

After weaning at approximately 4 months of age, lambs were moved from the experimental plots where they were reared to similarly treated areas of pasture (sludge-treated or control) and maintained there until slaughter at approximately 6 months of age.

We pooled subsamples taken from each delivered load of sludge for each half of the treated plot. The pooled samples (30 samples: 5 applications × 3 treated plots × 2 half plots) were analyzed for phthalate and alkylphenol content. Our methods and results from this portion of the study have been reported previously (Rhind et al. 2002). We treated control pasture with an equivalent amount of inorganic nitrogen applied in three lots during each application period to ensure that excessive amounts of nitrogen were not applied at any one time.

### Slaughter and tissue recovery.

In each of the 3 years of study, lambs from each treatment were slaughtered in an abattoir at approximately 6 months of age (November). Samples of liver, kidney fat, and muscle were recovered from each individual, wrapped in aluminum foil, and stored at –20°C until analysis. We also collected samples in each year from ewes that were at the end of their breeding life (cast for age). They were slaughtered at 5–6 years of age, having been exposed to the treatments for approximately 7, 19, or 31 months.

### Determination of tissue concentrations of nonlyphenol, octylphenol, and DEHP.

The EDCs in freeze-dried liver (0.4 g) were extracted into dichloromethane [20 cm^3^, containing 2.5 μg dihexylphthalate (prepared in-house) as an internal standard] at 50°C for 2 hr in sealed culture tubes on a dri-block heater. The mixture was cooled and filtered through Whatman no. 542 filter paper, and the filtrate was evaporated to dryness. The residue was dissolved in isohexane (4 cm^3^) and the solution washed on to an aminopropyl solid-phase extraction column with isohexane. After elution with diethyl ether (20 cm^3^), the eluate was dried under a stream of N_2_.

The EDCs in freeze-dried muscle (0.8 g) were extracted with dichloromethane (15 cm^3^ containing 0.45 μg S-1-phenyl-1-decanol (95% purity; Aldrich, Gillingham, UK) and 7.5 μg dihexylphthalate (prepared in-house as internal standards) at 50°C for 2 hr, in sealed culture tubes on a dri-block heater. The mixture was cooled, filtered through Whatman no. 6 filter paper and evaporated under a stream of N_2_ to 3 μL. We filtered the extract through a syringe filter (0.2 μm) to remove particulate matter and subjected the filtrate to gel permeation chromatography on an Envirosep ABC (350 × 21.2 mm) column (Phenomenex, Macclesfield, UK) using dichloromethane as the mobile phase to isolate EDCs from triacylglycerols.

To determine alkylphenols in kidney fat, we extracted the EDCs in freeze-dried kidney fat (1 g) with dichloromethane (15 cm^3^ containing 0.3 μg S-1-phenyl-1-decanol as internal standard) at 50°C for 2 hr, in sealed culture tubes on a dri-block heater. The mixture was cooled and filtered through Whatman no. 597 filter paper. The filtrate was evaporated to a small volume under a stream of N_2_ and passed through a syringe filter (0.2 μm) to remove particulate matter. We cleaned up the filtered material by passing it through an Envirosep ABC (350 × 21.2 mm) column (Phenomenex) and eluted the alkylphenols with dichloromethane.

### Determination of total phthalate in fat.

Fat (0.2 g), to which 1,2 phenylenediacetic acid [PDAA; 2.6 μg in 3 mL of 8% methanoic H_2_SO_4_ (vol/vol), 99% purity; Aldrich] was added as internal standard, was subjected to acid-catalyzed transesterification (90°C for 4 days). The reaction converted the phthalates to dimethylphthalate (DMP) and the internal standard to its dimethyl ester. After transesterification, we diluted the samples with water (10 cm^3^) and extracted them 2 times with isohexane (5 cm^3^). The extract was dried with anhydrous sodium sulfate, reduced to small volume under a stream of N_2_, and cleaned up on a silica column (2 rinses with 5 mL isohexane, followed by 2 rinses with 5 mL 5% diethyl ether/isohexane, vol/vol; the analyte was eluted with 3 rinses with 5 mL 20% diethyl ether in isohexane).

Several phthalates were likely to be present in the samples, but we measured only DEHP as being representative of this group of compounds; it is the phthalate ester that is produced in the largest amounts (Mylchreest et al. 2000). Although DEHP could be measured in extracts of liver and muscle, it was not possible to measure the concentration of any individual phthalates in fat because of the difficulty in isolating them from triacylglycerols and other glycacidic material.

### Measurement and quality control.

We determined EDC concentrations using gas chromatography linked to mass spectrometry (GC-MS) operated in the single ion recording mode. Two different instruments were used. In the first, a Fisons 8000 gas chromatograph fitted with an AS800 autosampler was coupled to a TRIO 1 quadrupole mass spectrometer (VG Masslab, Altrincham, Cheshire, UK). The second was a Trace DSQ gas chromatograph-mass spectrometer (GC-MS) system (Thermo Finnigan, Hemel Hempstead, UK) fitted with an AS 3000 autosampler. An HP5 fused silica capillary column [30 m × 0.25 mm (i.d.)] coated (0.25 μm) with 95% dimethyl/5% phenylpolysiloxane (Hewlett Packard Ltd., Stockport, UK) was operated with temperature programming (150–190°C at 7°C/min, to 200°C at 1°C/min and to 305°C at 10°C/min). For the analysis of methyl esters, the operating temperature of the column was 120°C for 1 min, 4.5°C/min to 190°C, and 15°C/min to 305°C. Helium was used as the carrier gas and samples were injected on to the GC column at a split ratio of 25:1.

The mass spectrometer was operated in the electron ionization mode (70 ev) and a source temperature of 200°C. The ions monitored for each compound were as follows: *m/z* 206 [octylphenol (OP)], *m/z* 135 [nonylphenol(NP)], *m/z* 150 (DEHP), *m/z* 149 (dihexyl phthalate), *m/z* 107 (phenyl decanol), *m/z* 163 (DMP) and *m/z* 190 (PDAA methyl ester). We calculated response factors relative to the internal standard for each component.

We extracted experimental blanks along with each batch of samples to ensure that the measured tissue concentration was not an artifact associated with any source of contamination. We calculated corrected tissue values by deducting the blank values from the measured values for each analyte.

Quality control was achieved by repeated analysis of a bulked sample of the appropriate tissue type. We calculated mean values and ranges (± 2 SD) for each of the analytes. These quality control samples were then included with each batch of experimental samples analyzed. The limit of detection for each of the analytes was 0.01 μg/g under these conditions. Data were accepted if there was< 10% variation between duplicate samples, and if not, we repeated the analysis.

### Statistical analysis.

Before statistical analysis, alkylphenol and phthalate concentrations were subjected to either square-root or log transformation to meet the requirement of constant variance.

Because in many year/treatment categories there were very few samples containing measurable amounts of NP, we included in the analysis only years with more than three samples per treatment in both treatment groups, and we present means based on untransformed data. All statistical analyses were conducted using Genstat (Lawes Agricultural Trust, 1994).

For ewe tissue, we used REML (residual maximum likelihood) analyses, specifying plot as a random effect, to assess the significance of treatment, year of slaughter, and associated interactions on concentrations in tissues. Within the REML analysis, effects of treatment, year, and interactions were assessed using approximate *F* tests.

For lamb tissue, a number of missing values in the data set invalidated the use of standard analysis of variance, necessitating the use of multiple linear regression to assess the significance of treatment, year of slaughter, sex of lamb, and associated interactions on concentrations in tissues. To allow for the incomplete balance in the structure of the data set, the main effect of any one of the above three factors was calculated after eliminating any effects of the other two factors. Likewise, the effect of interactions between any two factors was calculated after eliminating all other two-factor interactions. In practice, the required tests were obtained by carrying out the regressions a number of times, specifying the three factors of interest in different orders.

We calculated standard errors of differences in means using REML (Lawes Agricultural Trust 1994).

## Results

As reported previously (Rhind et al. 2002), NP and DEHP were present in large, but variable, amounts in the sewage sludge applied to the pastures, with concentrations in many subsamples > 100,000 μg/kg dry matter, whereas concentrations of OP were typically < 1,000 μg/kg dry matter (Table 1).

### Ewe tissue EDC concentrations.

Concentrations of OP in all tissues were either below the detection limit of the method or so close to it that statistical analysis was not meaningful. Measurable amounts of NP were present in few of the kidney fat samples, and so statistical analysis of these data was not conducted. In 2 of the 3 years studied, there were detectable levels of NP in liver and muscle samples, but for each tissue concentrations were below detectable levels in one of the years, although not in the same year (Table 2). Statistical analysis of the year effect was therefore confined to the 2 years in which NP was detectable. There were significant differences with year of slaughter in concentrations of NP observed in liver (*p* < 0.05) but not muscle.

Treatment effects were similarly inconsistent; mean concentrations were higher in control than in treated muscle tissue (*p* < 0.05), but there was no significant difference in liver concentrations. The highly variable patterns were reflected in significant treatment × year interactions with respect to muscle (*p* < 0.001) samples.

Mean liver and muscle concentrations of DEHP and fat concentrations of total phthalate (Table 3) were up to 100-fold higher than mean NP concentrations, but both differed markedly with year. The mean concentrations were higher in year 2 than in either years 1 or 3 in liver (*p* < 0.001), kidney fat (*p* < 0.01), and muscle (*p* < 0.001), and lowest in year 3. Concentrations in the tissues did not differ significantly with treatment (Table 3). However, there were significant interactions between treatment and year with respect to phthalate concentrations in liver (*p* < 0.01) and muscle (*p* < 0.001), and a similar trend in kidney fat. These interactions reflected the fact that concentrations in all three tissues were higher in treated than control ewes in years 1 and 3 (combined) in all tissues (*p* < 0.05 to *p* < 0.001) but exhibited the opposite trend in all tissues in year 2.

### Lamb tissue EDC concentrations.

We considered the low concentrations of OP found in lamb tissue unsuitable for statistical evaluation. Concentrations of NP in lamb liver and kidney fat were generally above the limit of detection, but because concentrations were close to the minimum limit detectable in most lamb kidney fat samples initially analyzed (year 2) and the extraction procedure was difficult and costly, we analyzed only six samples each for the remaining slaughter times, and we did not conduct statistical analyses on these data. Mean concentrations in liver and muscle differed significantly (*p* < 0.001) with year of slaughter, but the pattern was not consistent across tissues (Table 4). Mean concentrations were particularly high in treated animals in year 3, relative to other years and the control animals of that year. Mean concentrations were significantly higher (*p* < 0.001) in muscle from treated animals compared with control animals, but they were generally lower in other tissues and did not differ with treatment. Mean concentrations were similar in tissue from lambs of each sex. There were significant treatment × year interactions with respect to liver (*p* < 0.05) and muscle (*p* < 0.001), treatment × sex interactions with respect to liver (*p* < 0.05), and year × sex interactions with respect to liver (*p* < 0.05) and muscle (*p* < 0.01).

## Discussion

Associations between exposure to EDCs and physiologic disruption are well documented (Crisp et al. 1998; Toppari et al. 1996). Exposure to sewage and associated pollutants through contamination of drinking water has also been shown to be associated with reduced reproductive performance (Meijer et al. 1999) and altered behaviors of the offspring of ewes maintained on sludge-treated pastures throughout pregnancy and lactation (Erhard and Rhind 2004). However, understanding the possible involvement of EDCs in the induction of such effects depends on knowledge of levels of these EDCs in the tissues of the affected animal. Knowledge of tissue concentrations may also be pertinent to assessing risk to humans of exposure through the consumption of animal products.

Mean concentrations of NP and DEHP in the sewage sludge applied to the experimental plots were of a similar order of magnitude (approximately 50–250 mg/kg) and were comparable to levels reported previously in sewage sludges (Brunner et al. 1988; Webber and Lesage 1989) but were high relative to concentrations in soil, water, and air (Brooke et al. 1991; Rhind et al. 2002). Thus, the surface application of sludge to pastures has the potential to enhance the rate of accumulation of EDCs in the tissues of exposed animals relative to those maintained on conventionally managed pastures.

### Effects of class of pollutant on patterns of accumulation.

Whereas the concentrations of NP in sludge were similar to those of phthalates, NP concentrations in tissue were typically 10- to 100-fold lower than those of phthalate and were broadly similar to soil concentrations (Rhind et al. 2002), which are one measure of environmental concentrations. This may be attributable to rapid environmental degradation of alkylphenols after application to pasture (Rhind et al. 2002) rather than to differential metabolism or bioaccumulation after ingestion. We conclude that bioaccumulation of alkylphenols is unlikely to be a major concern with respect to the recycling of sewage sludge to pasture because most recorded tissue values were below the estimated no observed adverse effect level (NOAEL) of approximately 500 μg/kg, on a dry matter basis (Müller and Schlatter 1998).

Concentrations of DEHP in herbage, water, and air are generally very low and in soil may be low or undetectable (Brooke et al. 1991) or consistently above detectable levels (Rhind et al. 2002), as in the present study. The present study shows that, although environmental levels of phthalates are low, contrary to previous suggestions (Fries 1996), DEHP can accumulate in substantial amounts in ruminant tissues, with concentrations being approximately 10- to 30-fold higher than in the soil of the experimental pastures which were typically in the 10–20-μg/kg dry matter range (Rhind et al. 2002). These concentrations are at least as high as those reported in various fish species [13–86 μg/kg (wet weight); Brooke et al. 1991] and close to levels recorded in the fat of seals (10,600 μg/kg; Brooke et al. 1991), which, like humans, are near the top of the food chain.

These results highlight the importance of assessing the effects of DEHP and other EDCs in a range of species other than rodents because there are substantial species differences in the patterns of metabolism (Mylchreest et al. 2000) and therefore in bioaccumulation. Without detailed knowledge of the relationships between rate of uptake and degradation or excretion and of the biologic responses to exposure, it is impossible to assess the biologic significance of tissue concentrations of phthalates that were > 20,000 μg/kg in many tissues and individuals. However, at certain times of the production cycle, such as during pregnancy and lactation, fat tissue is mobilized in large amounts, releasing some of the EDCs stored in the tissue. Consequently, developing fetuses and neonates may be exposed to higher concentrations than those present in any tissue (Bigsby et al. 1997). Because daily dosing of rodents with phthalates, albeit at rates in the milligram per kilogram weight range, is known to result in adverse physiologic effects on the reproductive system (Davis et al. 1994; Mylchreest et al. 2000; Piersma et al. 2000), the potential impact on reproduction in domestic ruminants of the relatively high tissue concentrations observed in this study requires further investigation.

### Effect of sludge application on patterns of accumulation.

Use of sewage sludge as a fertilizer, even at the relatively high levels applied, was not associated with consistent increases in tissue concentrations of alkylphenols or phthalates in the animals maintained on the treated pastures. NP concentrations in many tissues and across years were at or below the detection limit, indicating that bioaccumulation was absent, and where concentrations were elevated, the effect was not consistently associated with exposure to sludge.

The higher concentrations of phthalate in each of the tissues of treated ewes relative to those of control ewes collected in years 1 and 3 appear to suggest that sludge application was associated with increased tissue concentrations of phthalate. However, the results of year 2, which were equally consistent across all tissues, although not always statistically significant, showed higher concentrations in control than treated animals, suggesting that the pattern of bioaccumulation can also be influenced by additional environmental factors. These could include differences in weather and pasture conditions or a major aerial deposition of phthalate, with associated changes in the pattern of exposure. The fact that the highest mean phthalate concentrations in each of the ewe tissues were also recorded in year 2 is consistent with the suggestion of an additional, major input of phthalate into the study area, as suggested previously (Rhind et al. 2002). If large enough, such an input could have masked, temporarily, the treatment differences observed in years 1 and 3. Because phthalate is rapidly degraded in soil (Rhind et al. 2002), such an effect would be transient.

The absence of evidence of higher levels of phthalate in most treated lamb tissues compared with control tissues may reflect the fact that the lambs had a different diet (primarily milk for the first 2 months of life) from the ewes and so were subject to different patterns of exposure. Alternatively, this may reflect the fact that the lambs were not exposed to the higher concentrations of phthalate present in the soil, and perhaps from other components of the environment, during winter (Rhind et al. 2002).

### Patterns of accumulation in different tissues.

It is frequently stated that EDCs are generally accumulated in fat tissue because they have lipophilic properties (Guillette et al. 1996). Although we noted accumulation of phthalate in fat tissue, there was also significant accumulation in muscle and liver. This may reflect the fact that membranes in all tissues contain lipids and so lipophilic chemicals can be expected to occur in any tissue. Even when different measures of phthalate concentrations used for kidney fat and other tissues are taken into account, it is clear that concentrations of phthalates in the three tissue types investigated were of a broadly similar order of magnitude. It could be argued, therefore, that analysis of a single tissue may provide an adequate measure of rates of accumulation for this class of chemical. In view of the complexity and cost of the analytical processes, this could be advantageous. Notwithstanding this suggestion, it is clear that similar patterns of accumulation in each tissue type must first be demonstrated, because this study showed some tissue differences in the pattern of EDC concentrations and certain tissues, such as testis tissue, exhibit a lower rate of accumulation of some EDCs (Cooke et al. 2001).

### Effects of duration of exposure, age, and sex.

We postulated that tissue concentrations of the selected EDCs would increase with increasing duration of exposure to sludge and associated exposure to EDCs. Consequently, higher concentrations were expected in ewe tissues than in lamb tissues. Similarly, ewes exposed to the sludge for a longer period were expected to have higher concentrations than those exposed for shorter periods. However, the results indicate that there was no consistent cumulative outcome of increased duration of exposure on the tissue concentrations of either of these classes of pollutants.

The fact that alkylphenol and phthalate concentrations did not increase consistently over time does not mean that the observed tissue concentrations, particularly of phthalates, were not biologically important. The effects of prolonged exposure of potentially susceptible tissues, such as may occur in species with a relatively long lifespan (including domestic ruminants used for the production of milk or offspring for meat, and humans) are uncertain (Suk et al. 2002). Furthermore, the release of chemicals during tissue mobilization, such as during pregnancy and lactation, may increase exposure (Bigsby et al. 1997), while interaction with other EDCs, even at very low concentrations, may greatly enhance effects (Rajapakse et al. 2002).

Although EDC concentrations in ewe and lamb tissues are not directly comparable, ewes are subject to more prolonged exposure and might be expected to exhibit higher tissue concentrations. In fact, tissue concentrations were generally as high, or higher, in lambs as in ewes, a finding consistent with the observation that tissue accumulation of these compounds is not merely a function of the duration of exposure. Factors that might be expected to affect accumulation include differences with animal age in their capacity to absorb, metabolize, distribute, or excrete the EDCs (Sjoberg et al. 1985) and differences in diet composition (milk and grass or grass only). In addition, the fact that the ewes had previously undergone several pregnancies and lactations means that they had undergone substantial mobilization of adipose tissue, probably with associated transfer to fetuses and milk of large amounts of stored EDCs, reducing the body burden of EDCs in the dams (Alcock et al. 2000) and transferring some of it to the offspring.

Overall mean tissue concentrations of phthalate were higher in the liver, but not muscle, of male compared with female lambs; concentrations were higher in female lambs in only one of the six treatment × year groups for each of these tissues. The causes of the sex difference in concentrations and of the difference between tissues in the consistency of the trends are unclear, but one possibility is a sex hormone-related difference in liver enzyme activity (Colby 1980). Regardless of the cause, these effects must be taken into account in future experimental designs.

## Conclusions

Exposure to sludge was associated with some increase in tissue phthalate concentrations, but the effect was not consistent and could apparently be masked by environmental factors. We conclude that the addition of sewage sludge to pasture is unlikely to induce large increases in tissue concentrations of NP and phthalates in sheep or other ruminants in comparable physiologic states grazing that pasture. The absence of an effect of exposure to sludge may reflect the fact that tissue DEHP concentrations associated with ambient exposure of control animals were relatively high.

## Figures and Tables

**Table 1:** Concentrations (μg/kg dry matter) of DEHP, NP, and OP in liquid sludge applied to the pastures (*n* = 30; 3 plots × 5 applications × 2 half plots).

	Mean ± SE	Range
DEHP	95,600 ± 6,500	58,300–208,600
NP	145,900 ± 10,600	73,500–283,500
OP	277 ± 140	< 1–3,680

**Table 2:** Mean concentrations (μg/kg dry matter) of NP in ewe tissues.

				SED		Significance		
	Year 1	Year 2	Year 3	Treatment	Year	Treatment	Year	Interaction
Liver*^a^*								
Treated								
No.*^b^*	11/15	4/14	2/12					
Concentration	7.45 (56)	4.36 (19)	— (45)	1.67	1.58	NS	*	NS
Control								
No.	13/14	7/19	2/16					
Concentration	10.57 (112)	7.30 (53)	— (265)					
Kidney fat								
Treated								
No.	1/15	0/14	2/12					
Concentration	— (45)	— (ND)	— (134)					
Control								
No.	6/15	3/18	3/16					
Concentration	— (77)	— (96)	— (29)					
Muscle*^a^*								
Treated								
No.	0/15	10/13	9/12					
Concentration	— (ND)	14.83 (220)	31.76 (1,009)	3.78	3.78	*	NS	#
Control								
No.	0/15	19/19	11/15					
Concentration	— (ND)	42.8 (1,832)	26.3 (692)					

Abbreviations: ND, not detectable, NS, not significant; SED, standard error of the difference. Data were square-root transformed (liver and muscle) before statistical analysis; back-transformed means are given in parentheses.

a Year 3 liver data and year 1 muscle data were not included in the statistical analysis because of low numbers of values above the detection limit.

b Numbers of samples which contained detectable amounts of NP/number assayed; for the purposes of statistical analyses, the detection limit was deemed to be halfway between zero and the minimum detectable value of 0.01.

* *p* < 0.05.

# *p* < 0.001.

**Table 3:** Mean concentrations (μg/kg dry matter) of DEHP (liver and muscle) or total phthalate (kidney fat tissue) in ewe tissues.

				SED		Significance		
	Year 1	Year 2	Year 3	Treatment	Year	Treatment	Year	Interaction
Liver								
Treated								
No.	15	14	12					
Concentration	73.4 (5,388)	54.3 (2,945)	28.4 (808)	6.75	8.26	NS	#	**
Control								
No.	14	18	16					
Concentration	40.3 (1,621)	73.7 (5,436)	23.3 (541)					
Kidney fat								
Treated								
No.	15	14	12					
Concentration	3.97 (9,333)	3.89 (7,762)	3.81 (6,457)	0.06	0.07	NS	**	NS
Control								
No.	14	18	16					
Concentration	3.80 (6,310)	3.99 (9,772)	3.67 (4,677)					
Muscle								
Treated								
No.	15	13	12					
Concentration	63.0 (3,969)	70.5 (4,970)	82.9 (6,872)	4.65	5.70	NS	#	#
Control								
No.	15	19	15					
Concentration	55.4 (3,069)	97.5 (9,506)	40.5 (1,640)					

Abbreviations: NS, not significant; SED, standard error of the difference. Data were log-transformed (kidney fat) or square-root transformed (liver and muscle) before analysis; back transformed means are given in parentheses. Detectable amounts of phthalate were present in all samples.

** *p* < 0.01.

# *p* < 0.001.

**Table 4:** Mean concentrations (μg/kg dry matter) of nonylphenol in tissues of male and female lambs.

	Year 1		Year 2		Year 3		SED			Significance		
	M	F	M	F	M	F	Treatment	Year	Sex	Treatment	Year	Sex
Liver												
Treated												
No.*^a^*	5/5	8/8	9/9	9/9	1/9	1/9						
Concentration	11.2 (125)	12.8 (163)	9.8 (97)	10.5 (111)	0.7 (0.44)	10.3 (107)	1.01	1.30	1.01	NS	#	NS
Control												
No.	6/8	7/8	9/9	8/9	1/9	9/9						
Concentration	6.9 (47.6)	6.7 (44.9)	13.6 (184)	11.0 (122)	1.4 (1.96)	4.0 (16.0)						
Kidney fat												
Treated												
No.	3/3	2/3	0/9	0/9	0/3	0/3						
Concentration	— (490)	— (7.2)	— (ND)	— (ND)	— (ND)	— (ND)						
Control												
No.	3/3	3/3	1/9	1/9	0/3	0/3						
Concentration	— (513)	— (347)	— (245)	— (510)	— (ND)	— (ND)						
Muscle												
Treated												
No.	8/9	4/8	8/8	6/9	9/9	9/9						
Concentration	13.0 (169)	9.15 (83.7)	16.7 (279)	21.5 (462)	43.5 (1,892)	37.9 (1,436)	1.85	2.23	1.85	#	#	NS
Control												
No.	3/8	5/9	9/9	9/9	9/9	9/9						
Concentration	10.6 (112)	10.4 (108)	7.07 (50.0)	18.7 (350)	22.7 (515)	18.3 (335)						

Abbreviations: ND, not detectable, NS, not significant; SED, standard error of the difference. Data were square-root transformed (liver and muscle) before analysis; back-transformed means are given in parentheses.

a Numbers of samples that contained detectable amounts of NP/number assayed; the limit of detection was 0.01 μg/g.

# *p* < 0.001.

**Table 5:** Mean concentrations (μg/kg dry matter) of DEHP (liver and muscle) or total phthalate (fat) in tissues of male and female lambs.

	Year 1		Year 2		Year 3		SED			Significance		
	M	F	M	F	M	F	Treatment	Year	Sex	Treatment	Year	Sex
Liver												
Treated												
No.	5	8	9	9	9	9						
Concentration	117.5 (13,799)	127.9 (16,348)	64.9 (4,224)	46.4 (2,157)	56.7 (3,211)	43.8 (1,919)	5.66	6.92	5.65	NS	#	#
Control												
No.	8	8	9	9	9	9						
Concentration	139.5 (19,452)	74.5 (5,547)	95.0 (9,027)	72.0 (5,181)	53.8 (2,899)	46.8 (2,194)						
Kidney fat												
Treated												
No.	9	9	9	6	8	5						
Concentration	4.14 (13,804)	3.79 (6,166)	4.13 (13,490)	3.80 (6,310)	3.58 (3,802)	3.53 (3,388)	0.052	0.064	0.052	NS	#	#
Control												
No.	9	9	8	8	9	9						
Concentration	4.32 (20,893)	3.66 (4,571)	4.06 (11,482)	3.94 (8,710)	3.52 (3,311)	3.57 (3,715)						
Muscle												
Treated												
No.	8	8	8	9	8	9						
Concentration	128.9 (16,615)	70.1 (4,914)	59.7 (3,564)	80.0 (6,400)	46.6 (2,172)	104.5 (10,920)	7.47	9.15	7.47	#	**	NS
Control												
No.	9	9	9	9	9	9						
Concentration	66.3 (4,396)	75.5 (5,700)	49.7 (2,470)	62.5 (3,906)	38.6 (1,482)	36.6 (1,340)						

Abbreviations: F, female; M, male; NS, not significant; SED, standard error of the difference. Data were square-root transformed (liver and muscle) or log-transformed (fat) before analysis; back-transformed means are given in parentheses. Detectable amounts of phthalate were present in all samples.

** *p* < 0.01.

# *p* < 0.001.
